# Associations between Student-Teacher Bonds and Oppositional Behavior Against Teachers in Adolescence: A Longitudinal Analysis from Ages 11 to 15

**DOI:** 10.1007/s10964-022-01645-x

**Published:** 2022-06-28

**Authors:** Sara Valdebenito, Lydia Speyer, Aja Louise Murray, Denis Ribeaud, Manuel Eisner

**Affiliations:** 1 Institute of Criminology, University of Cambridge, Cambridge, UK; 2 Department of Psychology, University of Cambridge, Cambridge, UK; 3 Department of Psychology, University of Edinburgh, Edinburgh, UK; 4 Jacobs Center for Productive Youth Development, University of Zurich, Zurich, Switzerland

**Keywords:** Student-teacher bond, Child-teacher relationship, Oppositional behavior, Cross-lagged effect

## Abstract

Prior research has found evidence for a positive effect of student-teacher bonds on children’s behavior. However, little research has investigated these relations following a transactional model of child development. This study investigated the bidirectional associations between student-teacher relationships and oppositional behaviors towards teachers using the ‘Zurich Project on the Social Development from Childhood to Adulthood’ (*n* = 1527; median ages 11, 13 and 15; 49% female). Results of a random-intercept cross-lagged panel model suggested that, among boys, positive student-teacher bonds at age 13 were associated with fewer teacher-reported oppositional behaviors two years later. The results indicated that negative interactions with teachers may be part of vicious cycles of poor relationships and increased levels of oppositional behavior, particularly for boys in late adolescence.

## Introduction

Positive student-teacher relationships have previously been found to increase children’s engagement with school learning and activity, improve academic performance and reduce the prevalence of children’s externalizing behavior (Roorda & Koomen, 2021). A transactional perspective of child development emphasizes the need to also investigate the impact of student behavior on the quality of student-teacher relationships as children actively participate in shaping their environment. However, such transactional processes have only received limited attention in the literature (e.g., Roskam et al., 2016). Adolescence, in particular, may be a key period for transactional associations between child-teacher relationships and adolescent behavior as, during this period, parental influence declines and relationships outside the family become increasingly more important (Nelson et al., 2016). Given the importance of externalizing behaviors in shaping student-teacher interactions as well as their impact on later developmental outcomes (e.g., Burke et al., 2014), this study thus examined the bidirectional dynamics of student-teacher bonds and students’ oppositional behavior against teachers from a transactional perspective.

According to attachment theory, children who display secure bonds to adults are more competent in social relationships, display more interest and skills in exploring new environments, and demonstrate less aggressive behavior than their insecurely attached peers (Flaherty & Sadler, 2011). While most research has focused on the role of parents as agents of socialization, recent findings have highlighted the importance of teachers for children’s developmental outcomes (Obsuth et al., 2016). For example, it has been observed that attachment to teachers increases children’s engagement in school (Flaherty & Sadler, 2011), has a positive impact on their academic outcomes (Roorda et al., 2011), and reduces the prevalence of children’s externalizing behavior (O’Connor et al., 2012). However, research not only points to the importance of student-teacher relationships for the development of children but also highlights the importance of children’s behavior for the wellbeing of teachers. Students’ behavioral problems have been found to be associated with high levels of teachers stress (Skaalvik & Skaalvik, 2015), burn-out (Aloe et al., 2014) and teacher turnover (e.g., Torres, 2014). In turn, teachers who manifest symptoms of stress and burn-out are more likely to be ineffective in teaching roles, provide less information and support, and increase the number of disciplinary referrals (Kokkinos, 2007), consequently affecting the quality of student-teacher relationships. These findings thus point to bidirectional associations between student-teacher relationships and students’ behaviors.

Importantly, such bidirectional associations are to be expected when following a transactional model of child development that views children as active participants in shaping their social environment (Roskam et al., 2016). Children’s behavioral development is thought to be partly influenced by the reactions they themselves evoke from key figures such as parents or teachers (Poulou, 2014). Thus, developmental outcomes do not emerge solely from the individual nor from the individual’s experience. Rather, outcomes are produced from the combination of an individual’s characteristics and their experiences and focusing singularly on one or the other to predict an outcome can be misleading. In the context of children’s success in the classroom, their behaviors and outcomes are arguably influenced by the school climate to which teachers significantly contribute. A number of cross-sectional studies have for instance highlighted the complex interplay between student and teacher characteristics in shaping the school environment. For example, in schools in which teachers had low expectations for their students, students perceived their teachers to be less supportive which in turn was associated with higher rates of school misconduct (Demanet & Van Houtte, 2012). Arguably, positive student-teacher relationships are a key aspect for shaping the school climate, having been found to be associated with a number of behaviors including reductions in bullying victimization and perpetration (Wang et al., 2015). In fact, evidence testing the transactional hypothesis shows that interchanges between students and teachers are organized in cycles of reciprocal influence (Ly & Zhou, 2016), thus, such transactions likely significantly contribute to shaping the school climate.

Behaviors that may be of particular relevance to the transactional dynamics in student-teacher interactions are externalizing symptoms such as aggression, oppositional defiant behavior and conduct disorder. Engaging in such behaviors likely elicits a direct response from an individual’s teacher and are the most frequent reasons that school students receive disciplinary referrals (Spaulding et al., 2010). Of particular relevance to student-teacher interactions, Oppositional Defiant Disorder (ODD) entails a recurrent pattern of negativistic, defiant, disobedient and hostile behavior directed towards authority figures (American Psychiatric Association, 2013), thus potentially explicitly evoking responses from any teacher to whom such behavior is directed. In the short term, patterns of oppositional and defiant behaviors can interfere with pupils’ acquisition of academic skills and subsequently reduce their attachment to the education system (Gottfredson et al., 2012). In the long term, ODD-related impairments have been linked with a range of later developmental difficulties (e.g., Burke et al., 2014) including involvement with the juvenile justice system (Pardini & Fite, 2010).

Studies testing bidirectional associations between child-teacher relationship quality and a child’s externalizing behaviors at school are scarce. While some studies have confirmed the bidirectional hypothesis, the available evidence on transactional associations is not consistent. A three-wave short-term longitudinal study of a kindergarten school year for instance found small bidirectional effects between children’s aggressive behavior at the beginning of kindergarten and an increase in child-teacher conflict at midyear (Doumen et al., 2008). conducted a. In turn, child-teacher conflict led to an escalation of aggressive behavior at the end of the year. Testing the transactional link between child-teacher relationships and behavioral adjustment in a sample of preschool boys at risk for developing externalizing problems, another study only identified bidirectional effects in waves 1 and 2 between conflict and externalizing behavior at the beginning of the school year and these effects were fairly small (Roorda et al., 2014). More recently a two-wave longitudinal study tested the bidirectional associations between child-teacher relationships and behavior problems in a sample of elementary Chinese-American immigrant schoolchildren (Ly & Zhou, 2016), finding transactional associations of a small effect size between the studied variables with teacher-rated externalizing problems negatively predicting child-rated closeness, and vice versa. Another study identified small effects based on a sample of Chinese nursery students (Zhang & Sun, 2011). Over a nine-month follow-up period, externalizing behavior at time 1 predicted teacher–child conflict at time 2. Reciprocally, teacher–child conflict at time 1 predicted externalizing behavior at time 2. Investigating the dynamic interplay between child-teacher relationship quality and 5-year-olds’ inattention and impulsivity, results of another study suggested that inattention and impulsivity at the end of kindergarten predicted less closeness with first-grade teachers (Portilla et al., 2014). However, conflict with teachers did not predict an increase in inattention and impulsive behaviors across time. Common to all of these studies was the focus on a relatively narrow age range primarily during the preschool years.

While the preschool years are an important period for the development of student-teacher relationships and externalizing behaviors, it is important to not only focus on this age range as such relationships may also be of key importance later during development. Adolescence is a particularly sensitive period for the social development of individuals as this is the time at which children start reducing their attachment to their parents and form increasingly more bonds outside the family (Nelson et al., 2016). The school environment plays a crucial role in adolescents’ transition into independence, ideally an *arena of comfort*, providing adolescents with social support in order to strengthen the young person to overcome other challenges in their life (Burke et al., 2014). If the school environment falls short of providing this arena of support, adolescents have to turn elsewhere. This is particularly problematic for adolescents as, unlike younger children who can usually rely on their home environment, adolescents likely try to find support arenas elsewhere, potentially in problematic environments for instance in the interaction with deviant peers. Arguably, positive student-teacher relationships represent a key aspect of the school environment being an arena of support. However, to date, relatively little research has investigated bidirectional associations between student-teacher relationships and children’s externalizing behaviors during adolescence. The little evidence that exists points towards student-teacher relationships having a protective effect on the development of externalizing behaviors (Wang et al., 2013), although this evidence is mixed (Murray et al., 2021; Obsuth et al., 2016). For instance, research studying ﻿the cross-lagged associations between student-teacher relationships and behavioral problems using a sample of 440 Finnish students (ages 10 to 14) showed that students who scored higher on externalizing problems were reported as more conflictive with teachers two years later (Pakarinen et al., 2017). More recently, another study found that aggression predicted poorer relationships with teachers for males across the ages 13 to 15 lag in males and across the age 11 to 13 lag in both genders (Murray et al., 2021).

One shortcoming of previous studies is that sample sizes were mostly modest, limiting subgroup analyses such as investigating the effect of gender. This is a limitation considering that developmental research has consistently found that student-teacher relationships differ by child gender. More specifically, female students generally present fewer conflictive behaviors than their male peers and more skills for cooperating and developing good relationships with school teachers (e.g., Mcgrath & Bergen, 2015). The most recurrent explanation for the differences in child-teacher relationships by students’ gender involves behavioral disparities. As consistently found in longitudinal studies (e.g., Ribeaud & Eisner, 2010), boys display more externalizing behaviors and more aggression than girls, which can translate into more conflicts with teachers as well as a higher likelihood of experiencing sanctions. For example, data by the Department for Education in England (DfE) suggested that male pupils are around three times more likely to be seriously punished than females (DfE, 2013). Similar trends have been observed in the US (Liu, 2013), Australia (Hemphill et al., 2010), Canada (Ministry of Education Ontario Canada, 2014) and the Netherlands (Coskun et al., 2015). A review suggested that teachers not only tended to give more warnings to boys than girls but also displayed a better rapport with girls and offered more academic support to them (Mcgrath & Bergen, 2015). Importantly, the role of gender may become increasingly relevant in interactions with teachers during adolescence. In particular, following gender intensification theory, socialization pressures during early adolescence lead to increasing adoptions of traditional sex-typed roles (Klaczynski et al., 2020), consequently impacting the relationships students establish with their teachers. Taken together, it is thus important to investigate gender differences when analyzing the bidirectional association between child-teacher bonds and oppositional behavior against teachers during adolescence.

A further limitation of most research on the bidirectional dynamics of the student-teacher relationship and externalizing behaviors is related to their use of teacher reports only (Portilla et al., 2014). When measurements of relationship and aggression are based exclusively on teacher ratings, this leaves open the possibility that ratings are biased by teachers’ implicit attitudes over and above the actual behavior of the children. This problem contributes to ‘shared method variance bias’ and refers to the fact that observed covariation can be inflated by the use of a common measurement method rather than the covariance of constructs the measures are expected to represent. Obtaining measures of the predictor and criterion variables from the same source is known to contribute to increased bias so that observed associations may be an artifact of rater effects (Maldonado-Carreño & Votruba-Drzal, 2011). Thus, it is important for further research to reduce shared method variance bias, through including independent reports from the two participants in this dyadic relationship (i.e., both teachers and students).

Finally, prior research has also been limited in its use of modeling strategies that conflate within- and between-person effects (Pakarinen et al., 2017). In particular, much of the longitudinal research relies on using methods such as the cross-lagged panel model that do not clearly separate within-person effects from between-person effects. That is, the effects of factors that differ between people may be related to both student-teacher relationships and externalizing behaviors, for example, stable aspects of the family environment or ethnicity (Hamaker et al., 2015). Considering that interventions aiming to reduce behavioral issues are aimed at the within-person level, it is highly necessary to conduct further longitudinal research using methods such as the Random-Intercept Cross-Lagged Panel model that disaggregates within- from between-person effects.

## Current Study

Previous research on child-teacher relationships and children’s developmental problems has primarily focused on investigating unidirectional associations, with most of the prior research focusing on investigating such associations during the preschool years. Given the importance of relationships outside the family during adolescence, the current study takes a transactional perspective and analyzes the bidirectional associations between student-teacher bonds and oppositional behaviors towards teacher in a sample of 1,527 adolescents taking part in the Zurich Project for the Social Development from Childhood to Adulthood (z-proso). Using random-intercept cross-lagged panel models that are well suited to overcome another limitation of prior research, that is the conflation of within- and between-person effects, this study investigated reciprocal effects across ages 11, 13 and 15, additionally testing for the effect of gender. It was hypothesized that a positive relationship between teachers and students reduces oppositional behavior against teachers and that the reduction in oppositional behavior can strengthen that positive bond between children and teachers. Further, it was hypothesized that such effects are stronger for boys than for girls given prior evidence for a heightened potential for student-teacher conflicts in boys.

## Methods

### Participants

The data were drawn from an ongoing prospective longitudinal study, the ‘Zurich Project on the Social Development from Childhood to Adulthood’ (hereafter ‘z-proso’). The study sample frame includes children who started in one of the 90 public primary schools in the City of Zurich during 2004. From this sample, 56 primary schools were randomly selected involving a target sample of 1,675 schoolchildren. Regarding demographic composition, 51.4% of the initial sample were male versus 48.6% female; 10.4% of the children were born outside Switzerland. Based on the children’s country of birth, 50% were non-Swiss. 27.1% of the sample reported divorced parents. Overall, the participants included in z-proso can be considered to be broadly representative of the city of Zurich’s youth population. In waves 1–4 the data were collected from the primary caregiver using computer-assisted face-to-face interviews. Data reported by children were collected using computer-assisted interviews (wave 1–3) and paper-and-pencil questionnaires (wave 4–7). In addition, in waves 1 through 7 children’s teachers were asked to complete assessments (i.e., paper-and-pencil questionnaires) providing information on children’s academic achievement, social behavior in the classroom and school problems. For details on the z-proso cohort, see the z-proso cohort protocol (Ribeaud et al., 2022).

This paper analyzes data from waves 4 to 6 of z-proso as these are the waves in which data on children’s oppositional behavior towards teachers and student-teacher bonds were collected. This comprises the age-years from Grade 5 (primary school) to the end of compulsory secondary school at Grade 9. At the age 11 wave, data was available for 1,139 adolescents, at the age 13 wave for 1,357 adolescents and at the age 15 wave for 1,444 adolescents. In total, 1,527 young people participated in at least one of the data collection waves used in the current study. For demographic characteristics of participants included in the current study, please see Table 1. Prior analyses of z-proso have suggested that dropout in the sample was related to primary caregivers speaking a minority language but not to adolescents’ behavior once analyses were adjusted for multiple comparisons (Eisner et al., 2019).

**Table 1:** Sample demographic information

Variable	Category	%	*N*
Child sex	Female	48.6	724
	Male	51.4	784
Country of birth	Switzerland	49.1	749
Female primary caregiver	Serbia-Montenegro	6.8	103
	Germany	3.7	57
	Portugal	5.8	88
	Italy	3.5	54
	Sri Lanka	5.4	82
	Other	25.8	393

		Mean	SD
Child age at data collection	Age 11 Wave	11.33	0.37
	Age 13 Wave	13.67	0.36
	Age 15 Wave	15.44	0.36

### Measures

#### Student-teacher bond (STB)

At ages 11, 13 and 15, students were asked to assess their relationship with their current teacher by rating the following three statements: “I get along with my teacher”; “my teacher is fair to me”; and “my teacher supports me”. Students responded to the items using a 4-point Likert scale ranging from “completely untrue” = “1” to “completely true” = “4”. If they had multiple teachers, participants were asked to give an average across all of their current teachers (teachers could change from year to year). A mean score of the responses for each item was utilized for descriptive analyses in the present study. At ages 11, 13 and 15 the composite variable yielded a Cronbach’s alpha equal to 0.78, 0.77, and 0.82, with 1,139, 1,357 and 1,444 students participating in the respective waves.

#### Oppositional behavior towards teacher (OBT)

Teachers reported data regarding students’ behavior at ages 11, 13 and 15 by using an adapted version of the Social Behavior Questionnaire (SBQ; Tremblay et al., 1992; Murray et al., 2019). A scale for measuring disruptive-oppositional behavior towards teachers was created using four items from the SBQ, namely “the child is disobedient”; “the child ignores the teacher”; “the child behaves impertinently”; and “the child disrupts the class”. Each item was rated based on a 4-point Likert scale from completely untrue = “1” to completely true = “4”. A mean score of the responses for each item was utilized for descriptive analyses in the current study. For ages 11, 13 and 15 the composite measures yielded Cronbach’s alphas equal to 0.90, 0.88 and 0.91. The numbers of teachers providing ratings at each measurement time were 274, 265 and 258, rating 1,036, 1,268 and 1,287 students respectively with students not necessarily being rated by the same teacher each year. Of the included teachers, 73% were female.

### Plan of Analysis

To examine the longitudinal relationships between student-teacher relationships and oppositional defiant behavior towards teachers, a Random-Intercept Cross-Lagged Panel Model (RI-CLPM) was fit using Mplus 8.8 (Muthén & Muthén, 2015). RI-CLPMs allow for the disaggregation of within- and between-person effects by estimating random intercepts before fitting an autoregressive/cross-lagged structure to the residuals (Hamaker et al., 2015). These random-intercepts are allowed to covary and thereby account for stable between-person effects that may be associated with both student-teacher relationships and oppositional defiant behaviors towards teachers. This specification thus allows the model to implicitly control for stable between-person covariates such as ethnicity or socio-economic status, making the explicit inclusion of such covariates in the model unnecessary (Speyer et al., 2022). Subsequently, the cross-lagged section of the analysis allows for the investigation of time-lagged associations between the two selected measures while controlling for the autoregressive pathways estimating the association between the within-person deviations of the variables at time *t* with time *t*_*+*1_.

In the current study, random intercepts were composed of latent variables resulting from a measurement model of student-teacher bonds and oppositional defiant behavior towards teachers. Specifically, a latent measurement model with strong factorial invariance was specified as invariance analyses suggested that longitudinal measurement invariance had not been substantially violated. For the cross-lagged part of the model, the effects of within-person changes in student-teacher bonds at time *t* on within-person changes in oppositional defiant behavior against teacher at time *t*_*+*1_ and vice versa were estimated. Finally, concurrent residual covariances between variables in the model at the time of assessment were included and allowed them to vary over time.

Model fit was assessed by the Tucker–Lewis Index (TLI), the Comparative Fit Index (CFI), the Root Mean Square Error of Approximation (RMSEA), and the Standardized Root Mean Square Residual (SRMSR). A good fit was indicated by values greater than or equal to 0.95 for TLI and CFI, less than or equal to 0.05 for RMSEA and less than 0.06 for SRMSR (Browne & Cudeck, 1992; Hu & Bentler, 1999; Pakpahan et al., 2017). To address the missing data, analyses used the Full Information Maximum Likelihood estimation (FIML) procedure. This procedure has proven to present less bias and greater efficiency than other conventional methods such as listwise or pairwise deletion (Wirtz et al., 2014). FIML provides unbiased parameter estimates provided that data are missing at random (MAR). Considering the non-normal distribution of some of the included variables, a robust maximum likelihood estimator was used (Rhemtulla & Savalei, 2012).

In addition to a combined model for boys and girls, a multi-group model was built to test for gender differences. In particular, a model with autoregressive and cross-lagged effects constrained to be equal across both genders was compared to a model where these effects were allowed to vary. If difference testing using the Satorra-Bentler scaled chi-square (Satorra & Bentler, 2009) suggested that the unconstrained model fit better than the constrained model, cross-lagged and autoregressive effects were also interpreted separately for each gender.

Full results of all analyses are available on the Open Science Framework (OSF): https://osf.io/2brv3/.

## Results

Tables 2 and 3 present correlations, means, and standard deviations of predictor and outcome variables. Overall, consistent with extant literature, z-proso data display a negative correlation between student-teacher bonds and oppositional behavior against teachers across all waves. Correlations range from *r* = −0.32; *p* < 0.01 in wave 4 (*M*_*age*_ = 11.32; SD = 0.36) to *r* = −0.28; *p* < 0.05 at wave 6 (*M*_*age*_ = 15.43; SD = 0.36). Furthermore, descriptive statistics suggested that while the overall mean of oppositional defiant behavior towards teachers stayed similar across the observed ages (Age 11 = 1.37, Age 13 = 1.31, Age 15 = 1.38) the means representing student-teacher bonds showed a small decrease from ages 11 to 15 (Age 11 = 3.47, Age 13 = 3.15, Age 15 = 3.06).

**Table 2:** Correlations, means, and standard deviations of variables involved in ARCL model

	1	2	3	4	5	6
1. OBT at age 11	1					
2. OBT at age 13	0.348**	1				
3. OBT at age 15	0.253**	0.416**	1			
4. STB at age 11	−0.322**	−0.118**	−0.095**	1		
5. STB at age 13	−0.168**	−0.253**	−0.186**	0.252**	1	
6. STB at age 15	−0.067*	−0.135**	−0.280**	0.150**	0.318**	1

*OBT* oppositional behavior towards teachers, *STB* student-teacher bond

*p < 0.05; **p < 0.01

**Table 3:** Means, standard deviation and paired-sample *t*-test

Behavioral variables	Full sample *M* (SD)	Range	Boys *M* (SD)	Girls *M* (SD)	*t* (*p* value)
OBT at age 11	1.37 (0.64)	1.00–5.00	1.51 (0.72)	1.21 (0.49)	7.74**
OBT at age 13	1.31 (0.58)	1.00–4.75	1.43 (0.65)	1.17 (0.45)	7.94**
OBT at age 15	1.38 (0.66)	1.00–5.00	1.49 (0.70)	1.25 (0.58)	6.55**
STB at age 11	3.47 (0.59)	1.00–4.00	3.39 (0.63)	3.55 (0.53)	−4.45**
STB at age 13	3.15 (0.65)	1.00–4.00	3.11 (0.66)	3.19 (0.63)	−2.21*
STB at age 15	3.06 (0.66)	1.00–4.00	3.03 (0.70)	3.09 (0.62)	−1.91

*OBT* oppositional behavior towards teachers, *STB* student-teacher bond

*p < 0.05; **p < 0.01

**Fig. 1:**
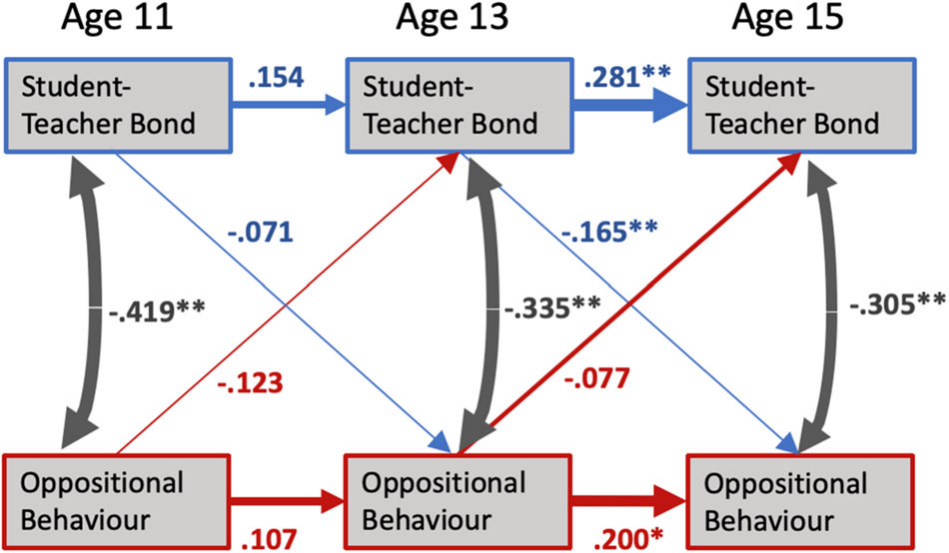
Overall autoregressive and cross-lagged param

The RI-CLPM fit for both boys and girls together provided a good fit to the data (CFI = 0.957, TLI = 0.954, RMSEA = 0.037, SRMR = 0.043). The autoregressive analysis demonstrated that oppositional behavior towards teachers and student-teacher bonds at age 13 significantly predicted oppositional behavior towards teachers and student-teacher bonds at age 15_._ Student-teacher bonds at age 13 further showed a significant negative cross-lagged effect on oppositional behavior towards teachers at age 15. In other words, results based on the full sample of students suggested that better child-teacher relationships at age 13 protect against the development of oppositional behavior towards teachers at age 15 (*β* = −0.165; *p* < 0.01). Results from the overall model are visualized in Fig. 1 and summarized in Table 4.

**Table 4:** Autoregressive and cross-lagged effects

	Overall		Girls		Boys	
Path	*β*	SE	*β*	SE	*β*	SE
Autoregressive						
OBT_11_ → OBT_13_	0.107	0.107	0.141	0.152	0.034	0.128
OBT_13_ → OBT_15_	0.200*	0.092	0.284*	0.121	0.127**	0.095
STB_11_ → STB_13_	0.154	0.086	0.081	0.128	0.340**	0.109
STB_13_ → STB_15_	0.281**	0.059	0.297**	0.081	0.452**	0.083
Cross-lagged						
OBT_11_ → STB_13_	−0.123	0.077	−0.201	0.128	−0.092	0.091
OBT_13_ → STB_15_	−0.077	0.061	−0.137	0.097	−0.020	0.072
STB_11_ → OBT_13_	−0.071	0.082	−0.206	0.136	−0.098	0.111
STB_13_ → OBT_15_	−0.165**	0.058	−0.106	0.075	−0.267**	0.095
(Residual) Covariances						
OBT_11_ with STB_11_	−0.419**	0.059	−0.496**	0.094	−0.308**	0.089
OBT_13_ with STB_13_	−0.335**	0.059	−0.348**	0.087	−0.359**	0.086
OBT_15_ with STB_15_	−0.305**	0.043	−0.250**	0.054	−0.341**	0.064
Model fitting						
RMSEA	0.037		0.048			
CFI	0.957		0.928			
TLI	0.954		0.925			
SMRM	0.043		0.069			

Parameters for girls and boys are based on an multi-group model with paths for autoregressive and cross-lagged effects as well as residual covariances being allowed to vary across the two groups

*OBT* oppositional behavior towards teachers, *STB* student-teacher bond

*p < 0.05; **p < 0.01

To examine the effect of gender suggested by previous literature (Jerome et al., 2009), first, a set of paired-sample t-tests was performed. Significant differences were revealed between boys and girls concerning student-teacher bonds as well as children’s oppositional behavior towards teachers across the years (see Table 3). Based on teachers’ assessments, at each wave, boys reported higher levels of oppositional defiant behavior towards teachers in comparison with girls; moreover, those differences were statistically significant for all ages. Girls displayed higher bonds toward teachers when compared with boys at age 11 and 13; at age 15 girls continued showing higher closeness to teachers than boys, but the difference in scores was not statistically significant.

After conducting descriptive tests, a multi-group model was fit to investigate the effect of gender on the observed autoregressive and cross-lagged effects, results of chi-square difference testing suggested that the unconstrained model indeed fit better than the constrained model (*χ*^2^(13) = 30.452, *p* < 0.001). Model fit statistics further suggested that the unconstrained model provided an acceptable fit to the data (CFI = 0.928, TLI = 0.925, RMSEA = 0.048, SRMR = 0.069). Comparing the results to the overall model fit on the whole sample, results suggested that the observed cross-lagged effect from student-teacher bonds at age 13 to oppositional behavior towards teachers only held for boys (*β* = −0.267; *p* < 0.01) but not for girls (*β* = −0.105; *p* > 0.05). Autoregressive effects were similar across both boys and girls aligning with the findings of the overall model. However, for boys an additional autoregressive effect for student-teacher bond across ages 11 to 13 was identified.

An RI-CLPM for boys and girls together without including a latent measurement model was run as a sensitivity analysis. The results of these analyses were substantially the same and they are available in full on the OSF: https://osf.io/2brv3/.

## Discussion

Previous research investigating the associations between student-teacher relationships and children’s behavioral issues have primarily focused on investigating unidirectional links even though transactional models of child development emphasize the importance of viewing children as active participants in shaping their environment and consequently also their teachers’ behaviors. In addition, prior research has mostly been limited by the use of cross-sectional data, small sample sizes and modeling strategies that conflate within- and between-person effects. The current study used three waves of data collected from 1,527 adolescents in Swiss schools to evaluate reciprocal influences between student-teacher bonds and oppositional behavior against teachers across ages 11, 13 and 15. The results offered limited evidence for reciprocal relations, only identifying an effect of student-teacher relationships on later student oppositionality in boys across the ages 13 to 15.

Findings demonstrated that student-teacher bonds and oppositional behavior against teachers were strong predictors of the same behavior two years later but only across ages 13 to 15, thus indicating positive within-person autoregressive effects across the sample in mid-adolescence. Generally, strong prediction of behavioral outcomes from past levels is not unexpected. The strongest predictor of behavioral or psychological outcomes is often their previous level of the same outcomes (Adachi & Willoughby, 2014). In fact, previous research testing similar bidirectional links with younger schoolchildren found that their measures of student-teacher conflict, aggressiveness, internalizing behavior and prosocial behavior were highly correlated with the earlier measure of the same outcome (Roorda et al., 2014). The fact that such autoregressive effects were only observed across ages 13 to 15 but not across ages 11 to 13 in the current study could be related to children in Switzerland transitioning from primary education to secondary education around age 12 (Swissinfo, 2022), thus potentially leading to discontinuity in their behaviors and relationships with teachers.

The cross-lagged parameters suggested support for a unidirectional link from positive student-teacher bonds at age 13 to fewer oppositional behaviors towards teachers at age 15. While this association was initially observed for the whole sample, analyses investigating the effect of gender suggested that the full-sample effect was driven by an effect in boys only. In other words, a better student-teacher bond among males predicted a small and significant decrease in oppositional behavior. The association was negative, significant with standardized coefficients below 0.30. It is important to emphasize that these rather small coefficients cannot be interpreted without considering the type of study design. The *β* coefficients produced by autoregressive cross-lagged models tend to be dramatically smaller than effects in cross-sectional studies for two reasons: 1) since behavior presents stability over time (which was the case in this study), the amount of change observed tends to be small; and 2) autoregressive models adjust for stability effects which in turn removes a large proportion of variance (Adachi & Willoughby, 2014). In the presented case, removing variance in oppositional behavior that is predicted by both gender and concurrent student-teacher bonds thus reduces the magnitude of the predictive effect of student-teacher bonds on oppositional behavior towards teachers two years later.

The effects reported in these analyses add evidence suggesting that a positive relationship with teachers can remain critical even during adolescence. Whereas the vast majority of research into transactions with teachers has previously focused on early childhood, the current study suggests that the effect remains into later phases of development. Since during adolescence individuals become more independent and in conflict with their parents, it may be that teachers can play more of a key role around this period of development than was previously assumed. Considering that adolescents go through a developmental stage when cognitive-control regions are still under development, the bond to teachers may be critical in supporting and influencing healthy patterns of behavior, prosocial decision making and socio-emotional learning. Leveraging changes at this stage can enable positive developmental trajectories not only in social but also in neurocognitive aspects (e.g., UNICEF Office of Research—Innocenti, 2017). Thus, findings of the current study are also consistent with previous evidence suggesting that positive intergenerational relationships can function as a protective factor for adolescents (Crosnoe et al., 2004). Importantly, it can be argued that this reduction in oppositional behavior is also a protective factor for teachers since student disciplinary problems such as violence, disrespect, misbehavior in school or oppositional behavior are associated with high levels of stress in teachers (e.g., Skaalvik & Skaalvik, 2015), burn-out (Aloe et al., 2014) and teacher turnover (e.g., Torres, 2014).

Taken together, these findings may have some important implications for schools and teachers. First, schools currently address children with disruptive/oppositional behaviors via interventions implemented in school settings to target children’s social skills, anger control and violence reduction. Even though these interventions show some level of impact, they could be accompanied by strategies focused on teachers’ skills and child-teacher conflicts and bonding. For instance, a recent randomized controlled trial, tested a low-cost intervention focused on encouraging teachers to adopt an empathetic attitude towards students (Okonofua et al., 2016). Results showed that this intervention reduced the number of school suspensions by 50% within a year. The intervention also contributed to enhancing respect between teachers and previously suspended students. Second, as observed in previous research (Doumen et al., 2008), the association between child-teacher relationships and oppositional behavior towards teachers show high autoregressive effects across ages 13 to 15. Accordingly, previous studies suggest that interventions aimed at improving the bond between students and teachers must start during the first years of schooling. Since findings suggest that student-teacher bonds can still have an impact on behavior during adolescence, early interventions should be enhanced by a sustained effort to reinforce a nurturing bond between both participants of this dyadic relationship. It may be particularly beneficial to focus on the transitional period from primary into secondary education as this may be a time of increased instability and disruption in student-teacher bonds.

Some limitations need to be considered when interpreting the results of the present study. First, in the current study, the variable capturing the student-teacher bond encompassed three items mostly referring to closeness to the teacher (i.e., “I get along with my teacher”; “the teacher is fair to me”; and “the teacher supports me”). Based on attachment theories, some previous studies have used at least two different constructs to measure student-teacher relationships: closeness, or the amount of positive affect in the relationship; and conflict, or the amount of discordance and anger in the relationship (O’Connor et al., 2012,). Moreover, a third concept that is dependency has been suggested, describing overly dependent and anxious behaviors of the child towards teachers (Roorda et al., 2014). Future research could benefit from the use of more comprehensive scales that may allow the analysis of different dimensions of student-teacher relationships. Another limitation to note is that cross-lagged models are sensitive to the time interval between waves, that is, in the current study, potential causal effects over two years. In comparison to most previous research, this is a comparatively large time-interval. Thus, some of the observed null-findings may be driven by the long time lag as the hypothesized relations may unfold over shorter periods (Dormann & Griffin, 2015). Also, it is important to mention that due to data availability limitations, only waves 4 to 6 (i.e., ages 11 to 15) were analyzed. It would be possible that transactional associations show a different trend at the earlier stages of development, hence, further research is needed.

Despite these limitations, the present study has several strengths. It used a large sample drawn from a multi-ethnic longitudinal study allowing for disentangling the reciprocal effect between student-teacher bonds and oppositional behavior for boys and girls separately. Further, in contrast with most previous studies, this study uses multiple informants in assessing student-teacher bonds and oppositional behavior against teachers, which reduces the risk of inflated estimates due to shared method variance bias. Finally, the study design used here is able to disaggregate within- and between-person effects. Thus, findings presented here offer some of the clearest evidence for the within-person associations between student-teacher bonds and oppositional behaviors towards teachers during adolescence to date.

## Conclusion

Even though transactional models of child development emphasize the importance of viewing children as active participants in shaping their environment and consequently also their teachers’ behaviors, previous research investigating the associations between student-teacher relationships and children’s behavioral issues have primarily focused on unidirectional links. Using a robust longitudinal study design that allowed for unambiguous insights into reciprocal within-person effects, the results of the current study suggested that positive student-teacher bonds may have a protective effect against the development of oppositional behaviors, particularly for boys. These findings emphasize the importance of positive student-teacher relationships during mid-adolescence with such relationships thus representing an important target for interventions aiming to reduce disruptive behaviors in the school context.
